# A Case of Hypoparathyroidism in Type 1 Diabetes Mellitus

**DOI:** 10.7759/cureus.37746

**Published:** 2023-04-18

**Authors:** Namra V Gohil, Aasvi V Gohil

**Affiliations:** 1 Internal Medicine, Medical College Baroda and Shri Sayajirao General (SSG) Hospital, Vadodara, IND; 2 Pediatrics, Gujarat Medical Education and Research Society (GMERS) Gotri, Vadodara, IND

**Keywords:** vitamin d, tremors, parathyroid hormone, diabetes mellitus, hypoparathyroidism

## Abstract

This case report discusses a 13-year-old female with a known history of type 1 diabetes mellitus presenting with pain in bilateral lower limbs, generalized weakness, and fatigue. After laboratory examinations, hypoparathyroidism was diagnosed based on low serum calcium, elevated serum phosphorous, and lower serum intact parathyroid hormone (PTH) levels. Treatment with calcium and vitamin D supplements led to a reduction in the patient's symptoms. The report provides an overview of the pathophysiology of hypoparathyroidism, its various etiologies, and clinical manifestations. The report emphasizes the importance of considering hypoparathyroidism as a differential diagnosis in patients with unexplained neuromuscular symptoms, even without a known thyroid disease or previous thyroid surgery.

## Introduction

Hypoparathyroidism is characterized by abnormally low or absent parathyroid hormone (PTH) synthesis or a problem with parathyroid hormone signaling. Low serum calcium and elevated serum phosphorus are the outcomes of this. Injury to the parathyroid glands or their unintentional removal during thyroid surgery is the most frequent cause of hypoparathyroidism [1]. Other rare causes of hypoparathyroidism are autoimmune (autoimmune polyglandular syndromes), infiltrative (hemochromatosis/Wilson's disease), certain genetic conditions, and hypomagnesemia.

## Case presentation

A 13-year-old known case of type 1 diabetes mellitus female presented with complaint of pain in bilateral lower limbs for three months. The pain was up to the knee, continuous initially and intermittent then onwards. The pain was mildly relieved with analgesics. The pain was sometimes associated with tingling and tightening sensation of the limbs. There was occasional stiffness and tremors in both feet. The patient also had complaint of generalized weakness and fatigue for two months. The patient had coarse and thin hair. There were no signs and symptoms of peripheral neuropathy except occasional tingling in both feet. There was no family history of diabetes mellitus or any other endocrine disorder. On examination the patient was vitally stable and systemic examination revealed no abnormality. The patient was examined for signs of latent tetany like Trosseau's sign and Chvostek's sign which were absent in this patient. Sensory and motor neurological examination was normal with normal tone, power and reflexes.

Clinically the following differential diagnoses were made: diabetic peripheral neuropathy, insulin neuritis and hypoparathyroidism. On laboratory examination her complete blood count, liver function tests, renal function tests and thyroid function tests were normal. Her laboratory investigations are mentioned in Table 1. 

**Table 1:** Laboratory Investigations

Investigation	Patient Value	Reference Range
Hemoglobin	14.6 g/dL	11-15 g/dL
Total Leukocyte Count	8600/mm^3^	4000-10000/mm^3^
Platelet Count	2,28,000/mm^3^	150000-410000/mm^3^
Serum Urea	20 mg/dL	14-40 mg/dL
Serum Creatinine	0.8 mg/dL	0.6-1.2 mg/dL
Serum Sodium	137 mEq/L	135-145 mEq/L
Serum Potassium	4.4 mEq/L	3.5-5.1 mEq/L
Total Bilirubin	0.8 md/dL	0.1-1.2 mg/dL
Serum Alanine aminotransferase	20 IU/L	0-40 IU/L
Serum Aspartate aminotransferase	32 IU/L	0-37 IU/L
Serum Alkaline Phosphatase	91 U/L	28-382 U/L
Serum Total calcium	7.8 mg/dL	8.5-10.5 mg/dL
Serum Ionised calcium	1.02 mmol/L	1.12-1.32 mmol/L
Serum Inorganic phosphate	>10 mg/dL	2.5-4.5 mg/dL
Serum Intact-Parathyroid hormone	10.64 pg/mL	15-65 pg/mL
Serum cortisol (morning)	18.2 microgram/dL	6.2-19.4 microgram/dL
Hemoglobin A1c	8.9%	<5.7%

Following the history, clinical examination and laboratory investigations, a diagnosis of hypoparathyroidism was made. The patient was given symptomatic treatment with analgesics: capsule tramadol (100 mg) 1-0-1 and pregabalin (75 mg) one tablet before bedtime for pain. She was given insulin for her diabetes. Following the diagnosis, she was given tablet calcium (500 mg) two tablets twice a day and oral vitamin D3 sachet (60,000 IU) once per day for seven days and then once a week. Patient’s lower limb pain, tremors and fatigue decreased following treatment.

## Discussion

PTH plays an important role in calcium and phosphate homeostasis. PTH acts directly in bone and kidney, but indirectly in the gastrointestinal tract by regulating renal 1,25-dihydroxy vitamin D [1,25(OH)2 D] production. Most neuromuscular symptoms and signs of hypoparathyroidism, such as tingling, numbness, paresthesias, and seizures, are caused by hypocalcemia, whereas hyperphosphatemia contributes significantly to ectopic mineralization in soft tissues (vasculature, brain, kidneys, and other organs) [2].

Idiopathic (genetic) hypoparathyroidism may occur alone or with other syndromic disorders. The autoimmune polyglandular syndrome type 1 is a syndromic form of hypoparathyroidism that happens with candidiasis and Addison's disease; DiGeorge syndrome (DGS) is a form that happens with immunodeficiency caused by thymic aplasia, congenital heart defects, and deformities; and the HDR anomaly, that happens with sensorineural deafness, renal cysts, and other renal deformities [3]. 

Pseudohypoparathyroidism (PHP) refers to a variety of metabolic disorders that are characterized by the presence of end-organ resistance to the action of PTH. The plasma concentrations of PTH are elevated in patients with pseudohypoparathyroidism. PHP is distinguished by its resistance to PTH, which can result from a variety of defects [4]. PTH resistance is caused by post-receptor defects such as PHP type 1A, PHP type 1B, PHP type 2, acrodysostosis types I and II, and PHP type 1c. In PHP 1A, GNAS mutations affect exons 1-13. In PHP 1B, there are GNAS methylation disturbances. In acrodysostosis types I and II, there are gene mutations that affect the regulatory subunit of protein kinase A and phosphodiesterase 4D, respectively. In PHP 1C, there are GNAS mutations that affect exon 13 [3,5,6]. Albright's hereditary osteodystrophy (AHO) is more frequently observed in PHP1A patients than PHP1B patients. AHO is characterized by the presence of short stature, obesity, round face, subcutaneous ossifications, reduced intellectual capacity, hypoplasia of dental enamel, hypogonadism, choroid plexus calcification, and short fingers and toes (brachydactyly) [3,7]. 

Current treatments involve taking calcium and active vitamin D supplements, with a goal of albumin-corrected serum calcium level in the range of 8-9 mg/dl. The activities of PTH are not replaced by this treatment, though, and it may result in both immediate issues (such as hypocalcemia, hypercalcemia, and increased urine calcium excretion) and long-term difficulties (which include nephrocalcinosis, kidney stones, and brain calcifications). Replacement with PTH is a new treatment option [8]. Indications to consider the use of recombinant parathyroid hormone(1-84) in hypoparathyroidism are as mentioned in Table 2 [9]. 

**Table 2:** Indications for considering the use of recombinant PTH(1-84) in hypoparathyroidism

Sr No.	Indication
1	Persistent hypocalcemia (serum total calcium <7.5 mg/dL) or persistence of symptoms of hypocalcemia
2	Oral calcium supplementation >2.5 g/day or 1,25-dihydroxy vitamin D >1.5 mcg/day or 1-alpha hydroxy vitamin D >3.0 mcg/day
3	Presence of hypercalciuria, nephrolithiasis, nephrocalcinosis, reduced creatinine clearance or estimated Glomerular Filtration Rate (<60 mL/min) or increased stone risk on urinary biochemical analysis
4	Persistent hyperphosphatemia (>4.5 mg/dL)
5	Gastrointestinal dysfunction
6	Reduced quality of life due to the disease

## Conclusions

Abnormal calcium and skeletal homeostasis are linked to hypoparathyroidism. It can be difficult to maintain control whilst using calcium and active vitamin D. The emergence of PTH(1-84) replacement therapy may open up new possibilities for improved control with fewer dietary supplementing needs.
